# Spinocerebellar ataxia type 14 (SCA14) in an Argentinian family: a case report

**DOI:** 10.1186/s13256-023-03897-y

**Published:** 2023-04-27

**Authors:** Niharika Duggirala, Kathie J. Ngo, Sabrina M. Pagnoni, Alberto L. Rosa, Brent L. Fogel

**Affiliations:** 1 Department of Neurology, David Geffen School of Medicine, University of California, Los Angeles, Los Angeles, CA 90095 USA; 2 Laboratorio de Genética y Biología Molecular, Fundación Allende Y Sanatorio Allende, Córdoba, Argentina; 3 Facultad de Ciencias Químicas, IRNASUS-CONICET, Universidad Católica de Cordoba, Córdoba, Argentina; 4 Department of Human Genetics, David Geffen School of Medicine, University of California, Los Angeles, Los Angeles, CA 90095 USA; 5 Departamento de Farmacología, IFEC-CONICET, Facultad de Ciencias Químicas, Universidad Nacional de Córdoba, Córdoba, Argentina

**Keywords:** Case report, PRKCG, SCA14, Spinocerebellar ataxia, Whole exome sequencing

## Abstract

**Background:**

Hereditary spinocerebellar ataxias are a group of genetic neurological disorders that result in degeneration of the cerebellum and brainstem, leading to difficulty in controlling balance and muscle coordination.

**Case presentation:**

A family affected by spinocerebellar ataxia was identified in Argentina and investigated using whole exome sequencing to determine the genetic etiology. The proband, a female white Hispanic aged 48, was noted to have slowly progressive gait ataxia, dysarthria, nystagmus, and moderate cerebellar atrophy. Whole exome sequencing was performed on three affected and two unaffected family members and revealed a dominant pathogenic variant, p.Gln127Arg (19:54392986 A>G), in the protein kinase C gamma gene, and the family was diagnosed with spinocerebellar ataxia type 14.

**Conclusions:**

To our knowledge, no previous cases of spinocerebellar ataxia type 14 have been reported in Argentina, expanding the global presence of this neurological disorder. This diagnosis supports whole exome sequencing as a high-yield method for identifying coding variants causing cerebellar ataxias and emphasizes the importance of broadening the clinical availability of whole exome sequencing for undiagnosed patients and families.

## Background

Cerebellar ataxia is a degenerative neurological disease primarily involving the cerebellum, which coordinates movement, leading to an inability to control one’s balance, eye movements, and voluntary muscle coordination [1, 2]. Age of ataxia onset varies significantly, from childhood to adulthood. Hereditary primary ataxias include nearly 50 forms of autosomal dominant spinocerebellar ataxias and over 30 autosomal recessive cerebellar ataxias [3–5]. Degenerative hereditary ataxias cause progressive degeneration of fine motor skills, including speech and balance [2, 6]. Genetic ataxias are characterized by many different mutation types including nucleotide repeat expansions, point mutations, and copy number variations. Over 500 genes associated with cerebellar ataxia have already been identified [7, 8]. Spinocerebellar ataxia 14 (SCA14) is an inherited autosomal dominant cerebellar ataxia, characterized by cerebellar atrophy and occasionally dystonia [9, 10].

In this study, we identified a multigenerational family from Argentina with a dominant from of cerebellar ataxia that remained undiagnosed after an initial round of genetic testing for the most common ataxic disorders. Next-generation clinical exome sequencing allows for a broader genome-wide evaluation of ataxia genes that can improve diagnosis rate following initial testing [7, 8, 11]. We describe the genomic analysis of this family of Argentinian descent in which three affected siblings presenting with spinocerebellar ataxia were found to have spinocerebellar ataxia type 14 (SCA14) due to mutation of the protein kinase C gamma (*PRKCG*) gene. To date, cases of SCA1, SCA2, SCA3, SCA6, and SCA7 have been reported in Argentina [12, 13] but, to the best of our knowledge, no prior cases of SCA14 have been reported in this region, expanding the global prevalence of this disorder.

## Case presentation

**Fig. 1:**
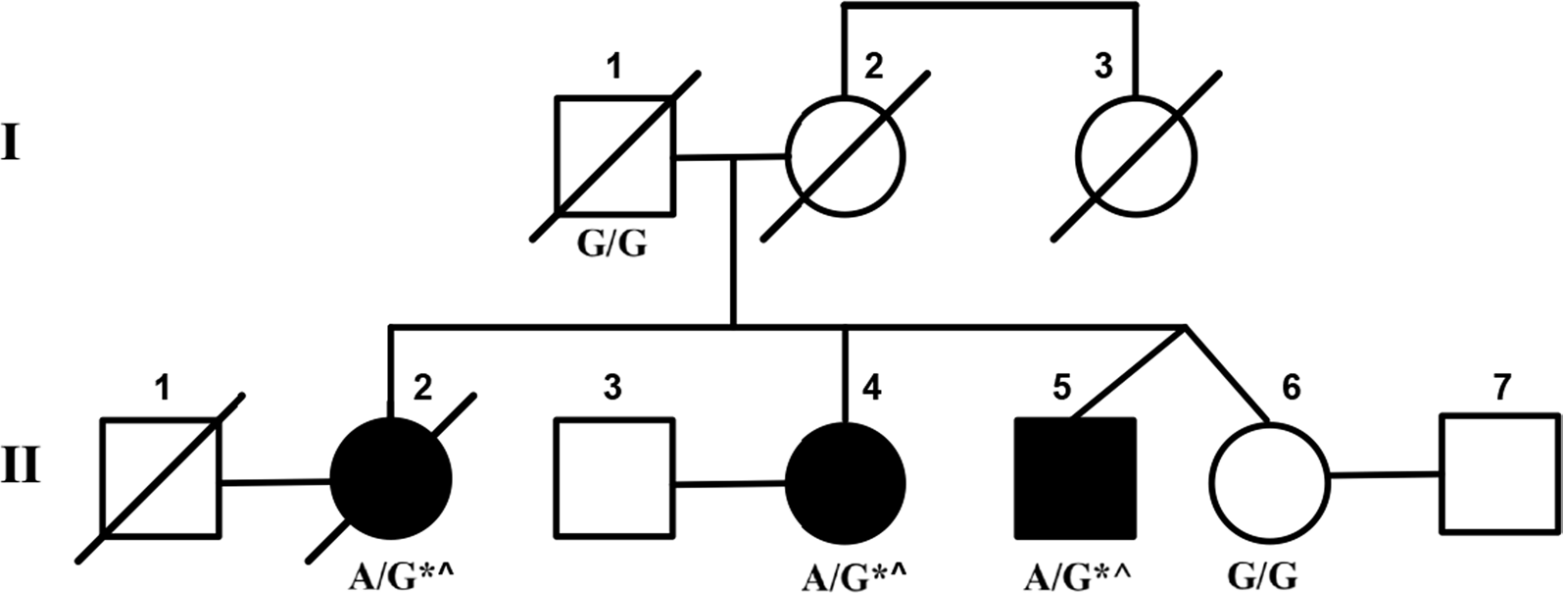
Pedigree. Index patient is individual II-2. Affected individuals are shown by shaded symbols. Deceased individuals are indicated by a line. Genotypes of the *PRKCG* gene are shown. G represents the reference while A is the p.Gln127Arg mutation. Affected family members and their corresponding phenotypes are indicated. *Gait ataxia, limb ataxia, horizontal nystagmus, ophthalmoplegia. ^Hyper-reflexia

A multigenerational family (Fig. 1) affected with cerebellar ataxia was evaluated. The proband, individual II-2, a white Hispanic female, was seen initially at age 48 and showed slowly progressive gait ataxia, dysarthria, nystagmus, and moderate cerebellar atrophy on brain magnetic resonance imaging (MRI) examination. Disease onset was at age 19 years, with all symptoms present by the fourth decade, and her condition remained slowly progressive until her death at age 62 years. Initial genetic testing for common dominant genetic ataxias was performed on individual II-2, and although this individual was negative for SCA1, SCA3, SCA6, SCA7, SCA17, and dentatorubral-pallidoluysian atrophy (DRPLA), an inconclusive result was obtained for SCA2.

Written informed consent was obtained from the family for participation in this study and publication of this case report. Both affected and unaffected family members consented for genetic analysis and this study was approved by the University of California, Los Angeles (UCLA) Institutional Review Board. Whole exome sequencing was performed and bioinformatically evaluated to assess for sequence and copy number variants as well as repeat expansions. Burrows-Wheeler Aligner was used to map the computed sequencing reads to the human genome [14]. Corresponding sequence alignment measures were quantified using Picard Tools [15], and repeated reads were marked. Variants were evaluated using VarSeq v2.2.1 (Golden Helix, Inc; http://www.goldenhelix.com). Variants were further filtered to exclude those with a minor allele frequency greater than 2% using the Genome Aggregation Database (gnomAD) public database [16]. This was due to the low likelihood of variants with a minor allele frequency greater than 2% causing Mendelian disease. Variants that had a disproportionately high allele frequency or allele count were eliminated from further annotation and classification. A list of keywords describing the patients and phenotypes that they presented with was then identified and used to produce a prioritized list of genes. This gene list was based on the clinical information found in the Online Mendelian Inheritance in Man database [17] and the Human Gene Mutation Database (HGMD) Professional Version [18]. Genetic variants detected within the genes in this list were evaluated initially prior to reviewing the remainder of the genomic variants. The HGMD and ClinVar [19] databases were used to assist annotation of these variants as pathogenic or likely pathogenic. Classification of variants was based on the guidelines provided by the American College of Medical Genetics and Genomics [20]. Detection of short tandem repeat (STR) expansions was performed using ExpansionHunter [21]. A Z score for each locus was calculated and used to determine how significant the number of repeats was by comparing it with the mean number of repeats at that locus. Loci of interest were filtered to only include those with a *Z*-score greater than two and were assessed on the basis of their segregation with disease status. Copy number variation (CNV) analysis was performed using WES data with the copy number inference from exome reads (CoNIFER) [22] and exome hidden Markov Model (XHMM) [23] tools. CNV calls were evaluated using the Database of Genomic Variants [24] and the DECIPHER [25] database based on reported pathogenicity and phenotypes.

Exome sequencing was initially performed on five family members of this multigenerational family, consisting of an unaffected parent, three affected siblings, and one unaffected sibling. As the family had previous inconclusive results when tested for SCA2, their exome data was further analyzed for repeat expansions using the ExpansionHunter tool, and no *ATXN2* repeat expansions were detected. No pathogenic copy number variants were detected following copy number variant analysis using the CoNIFER and XHMM tools. Sequencing analysis revealed a pathogenic variant in the *PRKCG* gene that was identified in all three affected siblings (Fig. 1). This *PRKCG* variant (19:54,392,986 A>G, p.Gln127Arg) is a single nucleotide missense variant not found in the gnomAD public database of human variation. This specific *PRKCG* variant has been identified as causing ataxia in multiple families, all with phenotypes consistent with spinocerebellar ataxia 14 [26]. Pathogenicity was determined using standard ACMG criteria (PS1, PS3, PS4, PM1, PM2, PP1, PP4) [20]. Clinical features of the examined family members (Fig. 1) are presented in Table 1. The general ataxia clinical phenotype in this family is consistent with the SCA14 phenotype [9, 10, 26].

**Table 1:** Clinical features of the Argentinian family

Individual	Age at onset	Age at examination	Initial symptom	Other symptoms	MRI	SARA
I-1	Unaffected	75 years	None	None	Not performed	0
II-2	17 years	48 years	Gait ataxia	Depression Dysarthria Hyper-reflexia Limb ataxia Nystagmus Ophthalmoplegia Pes cavus Proprioception loss Proximal amyotrophy	Cerebellar atrophy	28
II-4	19 years	39 years	Gait ataxia	Depression Dysarthria Hyper-reflexia Limb ataxia Nystagmus Ophthalmoplegia Pes cavus	Cerebellar atrophy	21
II-5	Unknown	37 years	Gait ataxia	Dysarthria Hyper-reflexia Limb ataxia Nystagmus Ophthalmoplegia	Not performed	12
II-6	Unaffected	37 years	None	None	Not performed	0

*MRI* magnetic resonance imaging, *SARA* scale for the assessment and rating of ataxia

**Fig. 2:**
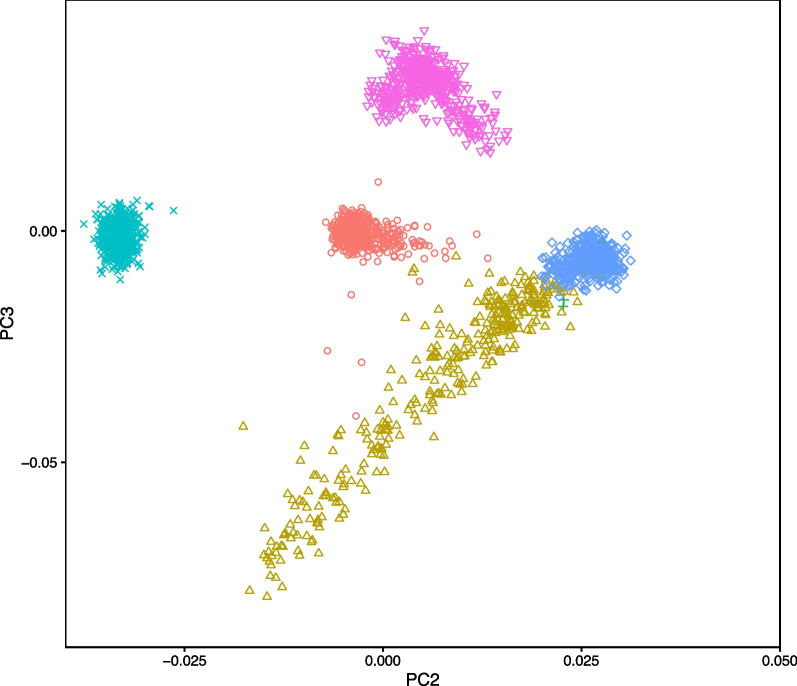
Genetic Ancestry Assessment. Plot shows the principal component analysis of the family in this report compared with subpopulations from the 1000 Genomes Project. *AFR* Africans (circle), *AMR* Admixed Americans (triangle), *ARG-FAM* Argentinian family in this report (+), *EAS* East Asians (x), *EUR* Europeans (diamond), *SAS* South Asians (inverted triangle)

To our knowledge, this is the first identified case of SCA14 in Argentina. However, it remained possible that ancestors of this family may have migrated to Argentina from other locations, such as Europe or Japan, where SCA14 is more common [27–32]. To assess the origins of this family, a genetic ancestry analysis was performed using principal component analysis of data from the 1000 Genomes Project. The resulting ancestry map was investigated to visualize clustering of this family with designated superpopulations. Although precise identification is not possible by this method, individuals from this family clustered most consistently with the Admixed American superpopulation (Fig. 2), as evidenced by the most nonsignificant *p*-value (0.34) and a *Z*-score most closely approaching zero (0.9467) (Table 2). The Admixed American superpopulation contains individuals from regions within South America, thus supporting ancestral origins of this family to this region of the world.

**Table 2:** Genetic ancestry assessment

Population	*p*-Value	*Z*-score
Admixed Americans	0.34	0.9467
Europeans	0.18	−1.3282
East Asians	0.13	−1.5096
Africans	0.07	−1.847
South Asians	0.03	−2.239

*p*-Values and *Z*-scores for the clustering of the family in this report with superpopulations from the 1000 Genomes Project are shown. *p*-Values closest to 1 and *Z*-scores closest to 0 reflect greatest similarity

## Discussion and conclusions

Exome sequencing was performed on an undiagnosed multigenerational Argentinian family affected by spinocerebellar ataxia. This revealed a pathogenic variant in the *PRKCG* gene, associated with spinocerebellar ataxia type 14 (SCA14). *PRKCG* encodes protein kinase C gamma, a serine/threonine kinase found in the Purkinje cells of the cerebellum that contributes to neuronal functions including synapse morphology, receptor turnover, and cytoskeletal integrity [33, 34]. The p.Gln127Arg identified in this family maps to the C1 regulatory domain of PRKCG, a common location for SCA14-causing mutations [33, 34]. This domain interacts with second messengers and regulates activation and membrane translocation of the protein [34]. Although the precise mechanism of pathogenesis remains unknown, one study has suggested that SCA14 mutations, including p.Gln127Arg, may promote amyloidogenesis [33].

SCA14-causing mutations in the *PRKCG* gene have been reported in Europe (including France, Germany, the Netherlands, and Scandinavia) and Japan [27–32]. Although multiple cases of SCA1, SCA2, SCA3, SCA6, and SCA7 in Argentinian families have been published [12, 13], to our knowledge this is the first example of SCA14 in this population. We further determined the ancestral origins of the family to be most consistent with the Admixed American superpopulation in the 1000 Genomes project data, thus expanding the prevalence of SCA14 to South America. This diagnosis emphasizes the importance and value of WES in identifying coding variants in cerebellar ataxias. WES has been recommended as a crucial step in the diagnosis of rare genetic disorders due to its broad efficacy [35], with a 17–35% success rate for neurological diseases [11] and a diagnosis rate of 25–50% for cerebellar ataxia [7, 8]. In this case, despite remaining undiagnosed following initial clinical and genetic testing, exome analysis obtained a definitive diagnosis. This underscores the importance of improving access to genetic diagnostic testing in developing countries that may have limited clinical resources or where access to such testing is impeded by cost to the patient. Access to WES could dramatically improve the diagnosis of rare genetic disorders affecting the populations in these locations. The cost of next-generation sequencing is rapidly decreasing and continued bioinformatic advances are anticipated to make this a cost-effective comprehensive single test for the diagnosis of genetically complex disorders such as ataxia [8, 36]. In developing countries, more modern technologies may be quickly adopted, in some cases by bypassing older technologies entirely [37], and this may be a potential means to closing this diagnostic testing gap. This study exemplifies the need for continuous research and analysis of modern next-generation sequencing methods and making them accessible to patients worldwide.

## Data Availability

The research data generated and analyzed in this study is not available or shared publicly to protect patient privacy but is available from the corresponding author on reasonable request.

## Abbreviations

CoNIFER: Copy number inference from exome reads
DRPLA: Dentatorubral-pallidoluysian atrophy
gnomAD: Genome Aggregation Database
HGMD: Human Gene Mutation Database
PRKCG: Protein kinase C gamma
SCA14: Spinocerebellar ataxia type 14
STR: Short tandem repeats
WES: Whole exome sequencing
XHMM: Exome hidden Markov Model
